# Left-handers know what’s left is right: Handedness and object affordance

**DOI:** 10.1371/journal.pone.0218988

**Published:** 2019-07-24

**Authors:** Nicole A. Thomas, Rebekah Manning, Elizabeth J. Saccone

**Affiliations:** 1 School of Psychological Sciences, Monash University, Melbourne, Australia; 2 College of Education, Psychology and Social Work, Flinders University, Adelaide, Australia; 3 School of Psychological and Public Health, La Trobe University, Bendigo, Australia; Universitat Wien, AUSTRIA

## Abstract

We live in a right-hander’s world. Although left-handers become accustomed to using right-handed devices, an underlying preference for objects that afford the dominant hand could remain. We employed eye tracking while left- and right-handed participants viewed advertisements for everyday products. Participants then rated aesthetic appeal, purchase intention, and perceived value. Left-handed participants found advertisements for products that more easily afforded them action to be more aesthetically appealing. They also indicated greater future purchase intention for products that were oriented towards the left hand, and gave these products a higher perceived value. Eye tracking data showed that object handles attracted attention, and were also able to retain participants’ attention. Further, across multiple eye movement measures, our data show that participant eye movements were altered by the orientation of the handle, such that this side of the image was examined earlier and for longer, regardless of handedness. Left-handers’ preferences might be stronger because they are more aware of object orientation, whereas right-handers do not experience the same difficulties. These findings highlight intrinsic differences in the way in which we perceive objects and our underlying judgments about those products, based on handedness.

## Introduction

Our handedness influences how we attend to, process, and interact with items in the physical world. Given that approximately 90% of the population is right-handed [1–4], we live in a world primarily designed by, and created for, right-handers [5]. As a result, left-handers need to adapt to this right-handed environment. Therefore, left-handers’ experience of the physical world must be quite different to that of right-handers. As everyday products, such as scissors, are specialised for use by either the left- or the right-hand, a market targeting left-handers exists. As popularised by *The Simpsons* [6], “leftorium” shops indeed exist in the UK, the USA, Australia, and, of course, online.

Gibson [7] proposed a relational perspective between the physical world and its perceiver. He suggested objects are perceived in terms of an individual’s ability to interact with them and introduced the term *affordance* as a way to describe possibilities for action in our environment. We are surrounded by affordances. For example, stairs afford climbing, and surfaces that are horizontal, flat, rigid, and sufficiently large afford support. Objects also afford actions; the handle of a teapot affords grasping. These possibilities for action exist independently of whether an individual recognises them or not; however, their perception is dependent on the individual’s capability and needs. As manual capabilities are different for left- and right-handers, it follows that handedness influences the perception of object affordances. Certainly, there is evidence that handedness affects how objects are processed and manipulated [8–10].

Although we are consciously unaware of it, affordances also influence object preferences, in that people prefer products that are easier to interact with [11–13]. For example, Eelen et al. [11] found right-handers reported higher preference ratings for right-handled objects. Interestingly, self-reported *flexible* right-handers showed a stronger preference for these products than *rigid* right-handers. (i.e., individuals who only ever use their right hand to manipulate objects). These findings suggest flexible right-handers pay more attention to object handle orientation than rigid right-handers. However, because Eelen et al. [11] did not test left-handers, we do not know whether they show a preference for left-handled objects.

Purchase decisions might also be influenced by handedness. Elder and Krishna [12] found participants showed greater intent to purchase a culinary dish with a utensil oriented toward the dominant hand than an equivalent dish with a utensil affording the non-dominant hand. Although this finding provides preliminary support for the idea that handedness influences purchase decisions, Elder and Krishna [12] used a single stimulus in each experiment, so whether their findings generalise across different objects is unknown. The authors also did not actively recruit left-handers, meaning their sample was predominantly comprised of right-handers. Therefore, it is once again uncertain whether a comparable bias in purchase decisions occurs amongst left-handers.

In the current study, we extended upon the findings of Eelen et al. [11] and Elder and Krishna [12] by employing a larger and more varied stimulus set to determine whether affordance-related preferences generalise across product types to reflect object valuation more generally. Furthermore, we recruited an equal number of left- and right-handed participants to determine whether equivalent preferences for products affording the dominant hand are seen across handedness groups. As left-handers interact with products designed for right-handers on a daily basis, left- and right-handers might not attend to and process affordances in an equivalent manner. In addition, we included a novel rating scale to assess perceived product value. This scale was used to determine whether left-handers, specifically, would attribute greater value to left-handed products.

A secondary goal of our research was to clarify the attentional processes that accompany product preferences. While handedness and object handle orientation appear to influence product ratings, the attentional mechanisms underlying these preferences remain unknown. It may be that object handle affordances are especially inclined to draw attention when they most easily afford action to the viewer; that is, when they are congruent with the dominant hand. Past research investigating whether handles are generally and inherently visually salient has produced inconsistent results on this matter [14–17]. Thus, it may be that motor-related preferences (e.g., handedness) modulate affordance-related attention, and that in turn, this attentional orienting predicts product evaluations. Eye tracking, which provides an objective measure of attentional orienting, was used to determine whether gaze patterns differ based on handedness and object handle orientation.

With respect to consumer research, Duchowski [18] suggested that eye tracking could provide insight into how consumers spread visual attention and which product features attract the most attention. For example, when examining visual scenes, items that have received a greater number of fixations are more strongly preferred than items that have not been fixated [19, 20]. Furthermore, when deciding between two alternatives, more fixations are directed toward the product that is ultimately chosen [21–25]. We examined whether handedness and object affordance interact to influence time to first fixation upon, total fixation duration, and the ordering of fixations within the left and right visual fields. An area of interest (AOI) was also created around the object’s handle to examine whether handle orientation and handedness interacted to influence scan patterns. As heat maps provide a visual summary of eye movements [26], we included separate heat maps for each advertisement to show which areas attracted the most attention.

We predicted handedness and product handle orientation would interact to influence participant judgments on all three rating scales. That is, right-handed participants would rate products as more aesthetically pleasing, report greater intent to purchase and attribute a higher monetary value to products with a right- than left-facing handle. For left-handers, we expected the reverse pattern.

With respect to eye movements, we predicted that participants would initially fixate upon the left side of the image, consistent with known tendencies to begin scanning on the left side amongst left-to-right readers. Furthermore, we hypothesised that participants would spend more time fixating upon the side of the image that contained the actionable part of the object (i.e., the handle). We also predicted handedness and handle orientation would interact to influence both time to first fixation and total fixation duration, such that we expected participants would fixate upon the side of the image that contained the object handle earlier and for longer when the handle was oriented toward, rather than away from, the participant’s dominant hand. In relation to our handle AOI, we expected that participants would fixate directly upon the handle earlier, and for longer, when it was oriented toward their dominant hand.

We also included an exploratory manipulation by showing half of our laboratory participants advertisements that contained text, whereas the other half of our laboratory participants saw advertisements that did not contain text. These advertisements were identical in all other facets. We chose to include this manipulation as we wanted to use advertisements that were highly ecologically valid (i.e., included text), but we also felt that the text might distract participants and cause them to spend less time fixating upon the objects overall. In this instance, we might have failed to observe significant differences in relation to eye movements simply because participants were focussed upon reading in lieu of examining the products themselves. As our rationale for including the advertisements without text was related to our eye movement data, we chose to manipulate this factor in our laboratory sample only. As such, we note that the majority of our participants saw advertisements that contained text. Therefore, we did not have any directional hypotheses in relation to advertisement type, but we included this factor within our analyses to determine whether the presence of text impacted eye movements.

## Method

### Participants

To estimate required sample size, we conducted an a priori power analysis (G*Power) assuming a small effect size (*d* = 0.20) for the between-participants analysis of variance (ANOVA) in relation to our ratings data. A sample size of at least 262 participants was needed to maintain 95% power at the .05 significance level. In order to achieve this sample size, we made use of two recruitment strategies. We recruited 100 undergraduate Flinders University students, who completed the experiment in exchange for $10.00AUD. All participants must complete basic pre-screen questions in order to access the University’s online system, which includes a question relating to handedness. As such, we were able to recruit participants based on their pre-screen handedness information without explicitly informing them that we were targeting left- and right-handers. These participants completed both eye tracking and ratings measures. To increase the number of responders for the ratings scales, we also recruited 176 individuals through the online crowdsourcing company, CrowdFlower. In this instance, we specifically recruited both left- and right-handed participants through the use of two surveys. These individuals received $1.50USD in exchange for their participation (see Pecher and van Dantzig [27], who also used online crowdsourcing to recruit left- and right-handed participants). All participants provided written informed consent prior to participation.

Our final sample consisted of 267 participants (156 males; *Mean*_*Age*_ = 32.45, *SD* = 9.84). Although we recruited 278 participants, as a result of a technical issue, eye tracking data failed to record for five Flinders University participants. Four online participants were also excluded, because they attempted to complete the survey twice. Handedness was determined using the FLANDERS handedness survey [28], which measures degree hand dominance. Based upon FLANDERS questionnaire scores, 124 participants were left-handed (*M* = -7.86, *SD* = 2.41) and 143 participants were right-handed (*M* = 9.04, *SD* = 2.11). Of these participants, 47 left-handers (48 right-handers) completed the experiment in the lab and 77 left-handers (95 right-handers) completed the experiment online. All participants had normal or corrected-to-normal vision. Ethical approval was granted by the Flinders University Social and Behavioural Research Ethics Committee (approval number: 6778).

### Apparatus

For individuals who participated in the laboratory, stimuli were presented on a Dell computer connected to a 17” Tobii T60 eye tracker (Tobii, Stockholm, Sweden), with a resolution of 1280 x 1024 pixels. Tobii Studio 3.3.1.757 software controlled stimulus presentation and recorded eye movements and participant responses. Prior to commencing, participants were aligned with the centre of the eye tracker to ensure the midsagittal plane and the eyes were in the centre of the screen. Distance to the screen was approximately 570 mm, but varied slightly (< 30 mm) between participants to obtain optimal eye tracking data.

### Stimuli

Images of 10 everyday objects (blender, detergent bottle, jug, kettle, mug, pot, saucepan, teapot, watering can) were chosen from Shutterstock’s online database. These objects were chosen because they were considered to be common and familiar. All images were mirror-reversed to generate left- and right-handled orientations of the products. These images were then used to create advertisements. Images were mirror-reversed prior to the creation of the advertisements and objects were positioned in the centre of the advertisement based on the midline of the entire object rather than the object’s body. We included text on the advertisements that referred to the products, which served to entice participants to buy the products, thereby more closely resembling actual advertisements. However, we also anticipated that the text on the advertisements would draw attention, leading participants to spend less time examining the products within the advertisements. Accordingly, advertisement type was varied between-participants in our laboratory sample only, such that half of the advertisements contained text, while the other half did not. Our online sample viewed advertisements with text only. Participants saw each of the 20 advertisements once in a pseudo-randomised order, such that the same object never appeared twice in a row. Each object was presented twice: once with a right-oriented handle and once with a left-oriented handle; although participants are likely to have noticed the repeated presentation of the objects, we do not believe participants would have systematically altered their ratings to be consistent with our hypotheses.

### Procedure

#### Laboratory

Following informed consent, participants completed a 9-point calibration on the eye tracker. Participants were tested alone, in a black cubicle, and the experimenter was positioned behind a black partition, out of the participant’s view. Participants were informed they would view a series of advertisements and they should scan these images freely, bearing in mind the following three questions: how aesthetically pleasing do you find the advertisement (*‘looks really good’*/*‘looks really bad’*); how likely would you be to purchase the advertised product (*‘extremely likely’*/*‘extremely unlikely’*); what is your perceived value of the advertised product (*‘$0’*/*‘$100’*). Each trial began with a fixation cross (1 second). Participants then saw an advertisement for a fixed time of 20 seconds, after which they completed the three rating scales. Participants were given an unlimited amount of time to register their ratings.

For each question, participants indicated their desired rating via mouse click on a horizontal visual analogue scale. We used visual analogue scales in order to detect smaller differences in ratings compared to a traditional 7-point Likert-type scale (i.e., [29]). As there are known leftward biases in Likert scales [30], and participants could have shown a bias towards the left end of the line [31], we used two versions of the rating scales. The label anchors of the scale were reversed so that half of the participants saw the ‘*looks really good*’, ‘*extremely likely*’, and ‘*$0*’ anchors on the left, whereas the remaining half saw these anchors on the right. The FLANDERS questionnaire was completed at the end of the experiment to avoid priming participants about handedness.

#### Online

Qualtrics online survey software was used to administer these stimuli on the CrowdFlower website on between June and September 2016. We used an extended sampling period to allow additional time for left-handed participants to complete the survey. Participation was voluntary and anonymous. Online instructions informed participants they would view a series of advertisements and should inspect each one freely and for as long as needed. Participants were informed that they would be asked to make 3 judgments for each advertisement: how aesthetically pleasing did you find this advertisement (‘*looks really good’/’looks really bad’*); how likely would you be to purchase this product (‘*extremely likely’/’extremely unlikely’*); what is your perceived value of this product (‘*$0’/’$100’*). Participants viewed advertisements for an unlimited amount of time while completing the rating scales. Similar, but slightly modified visual analogue scales were used to allow for online data collection. A bar was present in the centre of each scale and could be moved to the left or right to make judgements. The sliding scale produced scores between 0 and 100, but precise values were unbeknownst to participants, so small differences in ratings could be discerned. As above, the location of the label anchors was reversed for half of the participants. Participants then completed the FLANDERS [28].

### Statistical analyses

Deidentified data are available on the Open Science Framework, https://osf.io/d4pt7/

#### Ratings data

Ratings for aesthetic appraisal, purchase intention, and perceived value were recorded via cursor location on the visual analogue scale, resulting in an x-coordinate that was used to calculate scores. When the *‘looks really good’*/*‘extremely likely’*/*‘$100’* anchor was on the right side, judgments were determined by examining the x-coordinate, and converting this value into a percentage score. When these anchors were positioned on the left, x-coordinate values were subtracted from 720 (the total number of pixels on the visual analogue scale) to reverse the judgments, and then converted to a percentage. Percentage scores were used as they provide a more meaningful indication of where participants positioned their ratings along the visual analogue scale. Furthermore, this conversion meant that the online data, wherein possible values ranged between 0 and 100, and the lab data were comparable. Dollar values were calculated by dividing the x-coordinate by 720 and multiplying by 100 (maximum number of dollars). Likewise, ratings for aesthetic appraisal and purchase intention were divided by 720 and multiplied by 100, so values were on the same scale. As we manipulated the location of label anchors to control for any possible biases, we did not analyse this factor here; however, we have included an exploratory analysis in Supplementary Materials (S1 Text).

#### Eye tracking data

Saccades and fixations were distinguished using the Tobii Studio I-VT (velocity-threshold identification) fixation filter. Classifications were based on the velocity, in visual degrees per second, of directional shifts in eye movements [32]. Fixations were defined as eye movements with a velocity of less than 30 degrees (I-VT default value). Previous research shows that this default value is sufficiently accurate in distinguishing saccades and fixations, while accommodating noise in the data [33, 34].

**Fig 1:**
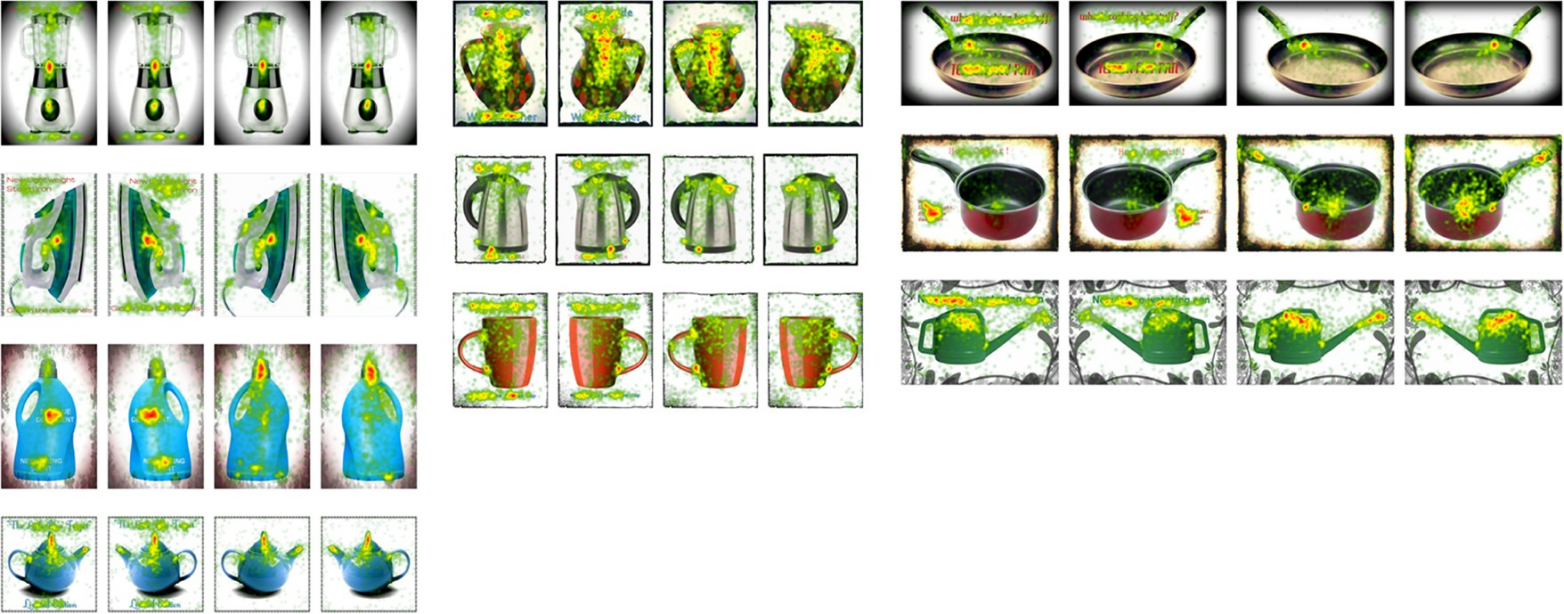
Heat maps depicting those areas of each advertisement that were fixated most often. All participants viewed both left- and right-handled objects and the heat maps show data collapsed across right- and left-handed participants. Red/green/yellow areas indicate where areas of the advertisements that were scanned by participants. Colours represent fixation frequency, progressing from red (most) to yellow to green (least). These colours clearly illustrate how the text on the advertisements influenced scanning patterns.

We created two overarching AOIs, right and left, to determine whether there were any horizontal asymmetries in eye movements. We were also interested to directly compare eye movements toward each object’s handle; as such, we created additional, more specific, AOIs for the handle of each object. For each of these AOIs, we calculated three dependent variables: time to first fixation (in ms), total fixation duration (in seconds), and fixations before (i.e., the number of fixations that preceded fixation upon the AOI–in this instance, the handle). We chose to use two measures of attentional attraction: one continuous (i.e., time to first fixation) and one count-based (i.e., fixations before), and one measure of attentional retention (i.e., total fixation duration), which allowed us to examine the attention-grabbing and attention-retaining properties of the advertisements separately. Lastly, we generated heat maps for each advertisement to show which areas received the most attention (Fig 1).

## Results

### Demographic data

**Fig 2:**
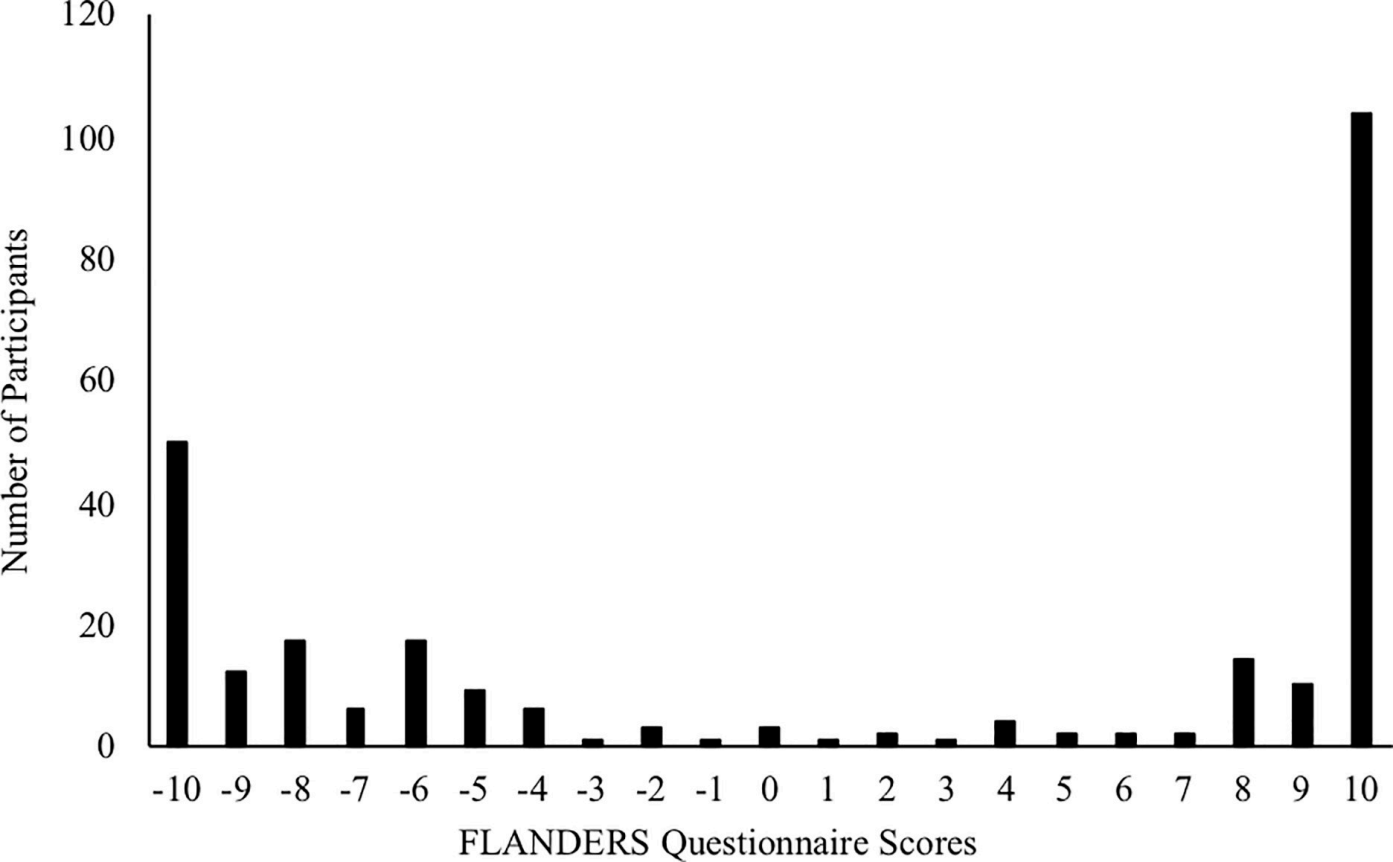
Frequency distribution of FLANDERS questionnaire scores for all participants.

As illustrated in Fig 2, our handedness data were more variable amongst left-handed participants, which is consistent with prior research [35, 36]. In the interest of examining whether strength of handedness differentially influenced performance on the rating scales, we conducted a series of Pearson product-moment correlations. We calculated difference scores for all rating scales wherein negative scores reflected a preference for advertisements of objects with handles on the left side, whereas positive scores indicated a preference for advertisements with right-handled objects. Intriguingly, all three rating scores were positively correlated with handedness scores. Participants who showed stronger hand preferences also showed stronger preference scores on the ratings scales for advertisements where the handle was oriented toward the dominant hand (aesthetics: *r*(267) = .189, *p* = .002, purchase intention: *r*(267) = .209, *p* = .001, perceived value: *r*(267) = .231, *p* < .001).

### Rating scales

We conducted 2 (handedness: left, right) x 2 (handle orientation: left, right) x 2 (advertisement type: text, no text) ANOVAs for each rating scale: aesthetic appraisal, purchase intention, and perceived value.

#### Aesthetic appraisal

**Fig 3:**
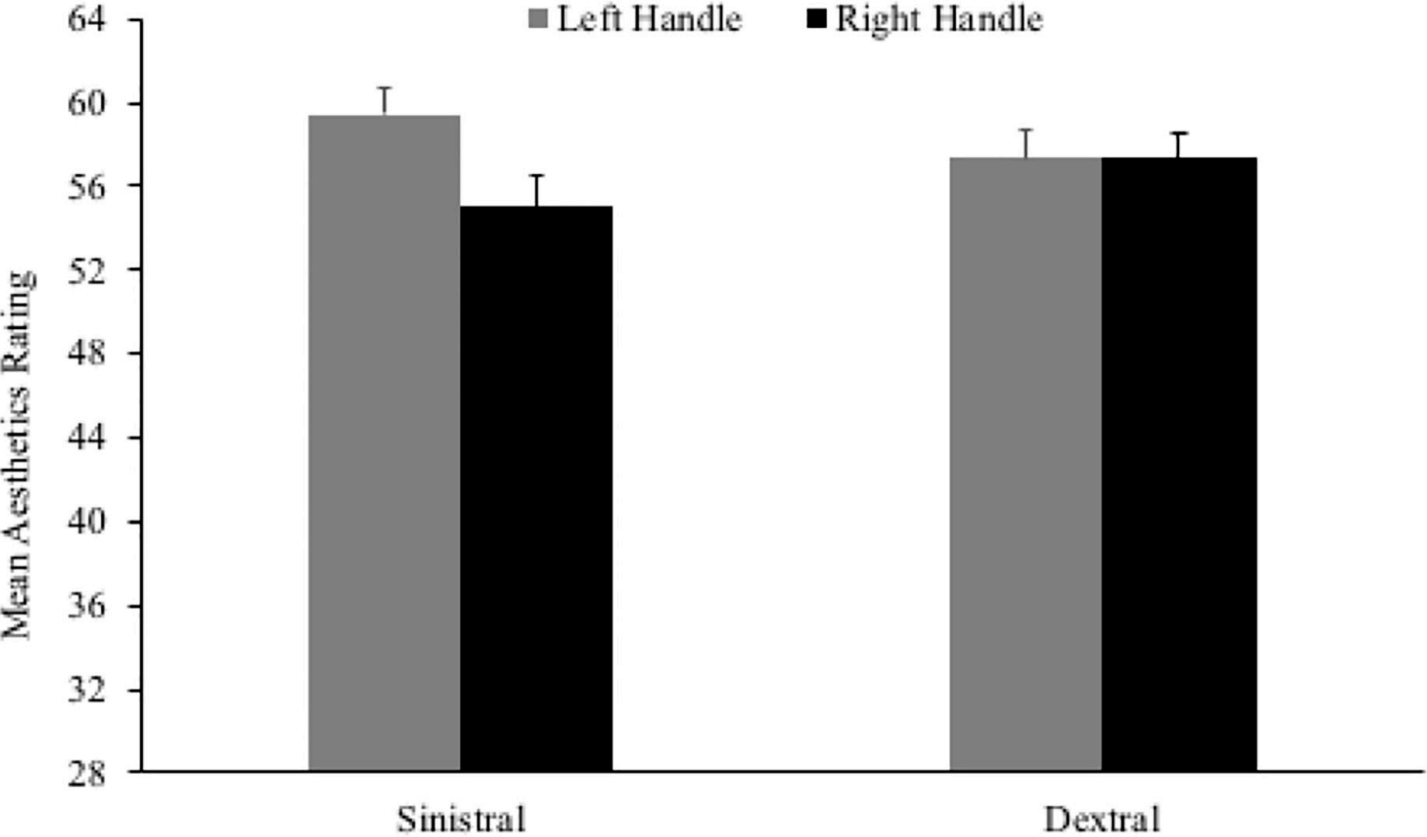
Mean aesthetic appraisal for left- and right-handled objects across left- and right-handed participants.

The main effect of handedness, *F*(1,263) = .000, *p* = .989, $\eta_{p}^{2}$ < .001, was non-significant, indicating that overall ratings did not differ based on handedness alone. However, the main effect of handle orientation was significant, *F*(1,263) = 5.349, *p* = .022, $\eta_{p}^{2}$ = .020. Overall, mean aesthetics ratings were higher for advertisements where products had the handle on the left side, compared to the right. Importantly, the predicted interaction between handle orientation and handedness was significant, *F*(1,263) = 5.718, *p* = .017, $\eta_{p}^{2}$ = .021 (Fig 3). Paired-samples *t*-tests showed that left-handers rated advertisements as more aesthetically pleasing when the handle was on the left compared to the right, *t*(123) = 4.138, *p* < .001, *d* = .375. In contrast, right-handers gave similar ratings to advertisements, regardless of handle orientation *t*(142) = .013, *p* = .990, *d* = .000. None of the effects in relation to advertisement type were significant: advertisement type, *F*(1,263) = .518, *p* = .472, $\eta_{p}^{2}$ = .002; handle orientation x advertisement type, *F*(1,263) = 1.446, *p* = .230, $\eta_{p}^{2}$ = .005; handedness x advertisement type, *F*(1,263) = .015, *p* = .902, $\eta_{p}^{2}$ < .001; handle orientation x handedness x advertisement type, *F*(1,263) = 1.148, *p* = .285, $\eta_{p}^{2}$ = .004.

#### Purchase intention

**Fig 4:**
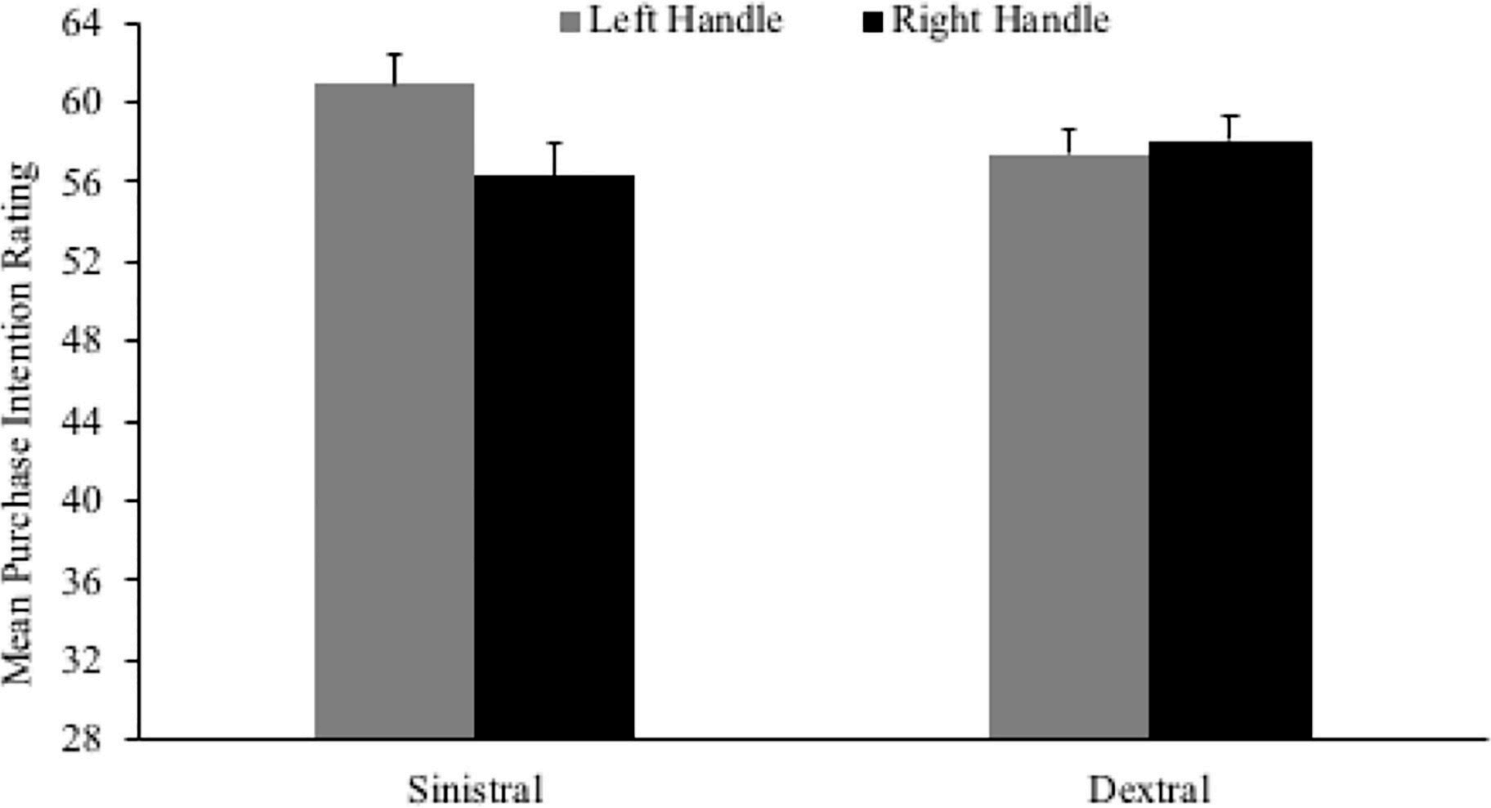
Mean purchase intention for left- and right-handled objects across left- and right-handed participants.

In contrast to the findings in relation to aesthetic appraisal, the main effect of advertisement type was significant for purchase intention, *F*(1,263) = 14.870, *p* < .001, $\eta_{p}^{2}$ = .054. Participants indicated higher future purchase intention for advertisements without text (*M* = 65.97, *SD* = 15.41), as compared to those with text (*M* = 56.42, *SD* = 15.48). Although the main effects of handedness, *F*(1,263) = .316, *p* = .574, $\eta_{p}^{2}$ = .001, and handle orientation, *F*(1,263) = 2.750, *p* = .098, $\eta_{p}^{2}$ = .010, were non-significant, the critical interaction between handle orientation and handedness was once again significant, *F*(1,263) = 10.287, *p* = .002, $\eta_{p}^{2}$ = .038 (Fig 4). Paired-samples *t*-tests illustrated that left-handers indicated higher future purchase intention for advertisements where object handles were on the left, as compared to the right, *t*(123) = 4.648, *p* < .001, *d* = .419. In contrast, right-handers indicated similar future purchase intention for both advertisements, *t*(142) = 1.279, *p* = .203, *d* = .107. Remaining interactions were not significant: handle orientation x advertisement type, *F*(1,263) = 3.110, *p* = .079, $\eta_{p}^{2}$ = .012; handedness x advertisement type, *F*(1,263) = .219, *p* = .640, $\eta_{p}^{2}$ = .001; handle orientation x handedness x advertisement type, *F*(1,263) = .839, *p* = .361, $\eta_{p}^{2}$ = .003.

#### Perceived monetary value

**Fig 5:**
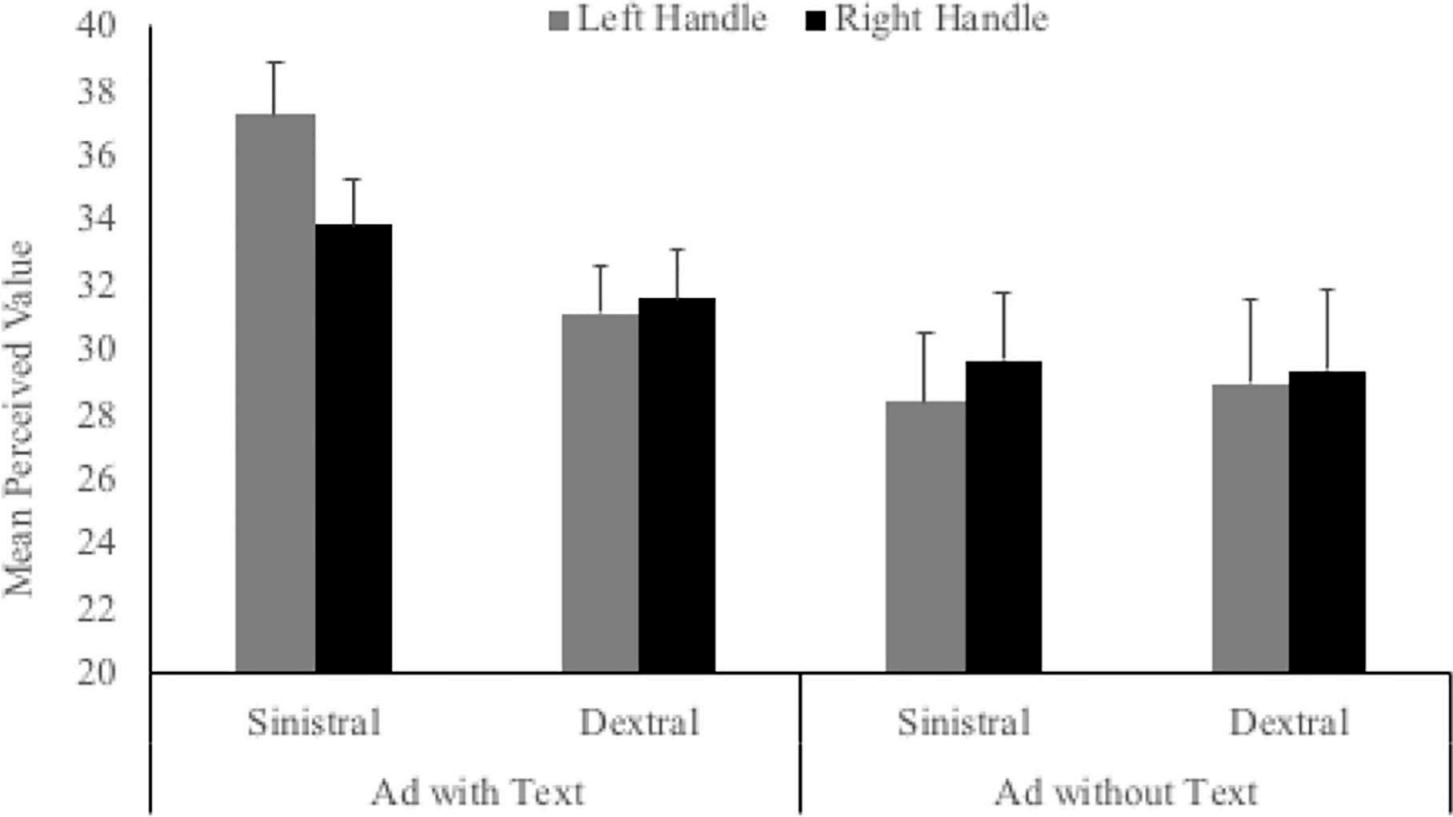
Mean perceived monetary value ($AUD) for left- and right-handled objects across left- and right-handed participants.

The main effects of handedness, *F*(1,263) = .744, *p* = .389, $\eta_{p}^{2}$ = .003, handle orientation, *F*(1,263) = .332, *p* = .565, $\eta_{p}^{2}$ = .001, and advertisement type, *F*(1,263) = 3.448, *p* = .064, $\eta_{p}^{2}$ = .013, did not reach significance. In contrast to the first two rating scales, the critical interaction was not significant, *F*(1,263) = 1.900, *p* = .169, $\eta_{p}^{2}$ = .007 (Fig 5). The interaction between advertisement type and handedness was non-significant, *F*(1,263) = .863, *p* = .354, $\eta_{p}^{2}$ = .003; however, the interaction between handle orientation and advertisement type was significant, *F*(1,263) = 4.718, *p* = .031, $\eta_{p}^{2}$ = .018, and was qualified by a significant 3 way interaction, *F*(1,263) = 4.660, *p* = .032, $\eta_{p}^{2}$ = .017.

To explore the significant 3-way interaction, data were split according to advertisement type. For advertisements with text, the main effects of handle orientation, *F*(1,217) = 8.928, *p* = .003, $\eta_{p}^{2}$ = .040, and handedness, *F*(1,263) = 4.093, *p* = .044, $\eta_{p}^{2}$ = .019, were both significant. The interaction was also significant, *F*(1,217) = 14.786, *p* < .001, $\eta_{p}^{2}$ = .064 (Fig 5). Paired-samples *t*-tests showed that left-handed participants indicated a higher perceived value for advertisements where object handles were on the left compared to the right, *t*(99) = 3.461, *p* = .001, *d* = .353. Right-handed participants gave similar perceived value ratings for both advertisements, *t*(118) = 1.097, *p* = .275, *d* = .103. In contrast, for advertisements without any text, none of the effects reached significance: handle orientation, *F*(1,46) = 3.857, *p* = .056, $\eta_{p}^{2}$ = .077; handedness, *F*(1,46) = .002, *p* = .963, $\eta_{p}^{2}$ < .001; handle orientation x handedness, *F*(1,46) = .922, *p* = .342, $\eta_{p}^{2}$ = .020. As our sample was significantly smaller for the advertisements without text, we likely did not have enough power to detect significant effects in this subsample. As such, the lack of differences in perceived value ratings for advertisements without text should not be over interpreted.

### Eye tracking data

We examined whether fixation patterns differed based on handedness and object affordance. Our dependent variables were time to first fixation, total fixation duration, and fixations before. We conducted 2 (handle orientation: left, right) x 2 (visual field: left, right) x 2 (handedness: left, right) x 2 (advertisement type: text, no text) mixed-model ANOVAs for our three dependent variables. In addition, we used paired-samples *t*-tests, with a Bonferroni-corrected α = .013, to compare eye movements toward the handle, based on handedness and object affordance.

#### Time to first fixation

As none of the effects in relation to advertisement type were significant, we repeated the analysis with this factor removed to increase our power. There was a main effect of visual field, *F*(1,93) = 10.873, *p* < .001, $\eta_{p}^{2}$ = .105, which was qualified by an interaction with handle orientation, *F*(1,93) = 9.673, *p* = .002, $\eta_{p}^{2}$ = .094. Remaining effects were non-significant (Table 1), indicating handedness did not influence time to first fixation. The main effect of visual field showed that participants examined the left visual field (*M* = 1.96 s, *SD* = .94) earlier than the right visual field (*M* = 2.29 s, *SD* = .89). The interaction of visual field and handle orientation showed that fixations occurred earlier within the left visual field, as compared to the right, when the object handle was located on the left side, *t*(94) = 4.548, *p* < .001, *d* = .476. Furthermore, fixations within the right visual field occurred earlier for advertisements of right handled objects, compared to left handled objects, *t*(94) = 3.732, *p* < .001, *d* = .376. Remaining comparisons were non-significant (Table 1).

**Table 1:** Statistics for time to first fixation.

Effect	Statistic
Handle Orientation	*F*(1,93) = 2.294, *p* = .133, $\eta_{p}^{2}$ = .024
Handedness	*F*(1,93) = .006, *p* = .941, $\eta_{p}^{2}$ < .001
Handle Orientation*Handedness	*F*(1,93) = .182, *p* = .670, $\eta_{p}^{2}$ = .002
VF*Handedness	*F*(1,93) = .017, *p* = .897, $\eta_{p}^{2}$ < .001
Handle*VF*Handedness	*F*(1,93) = 1.876, *p* = .174, $\eta_{p}^{2}$ = .020
Left Handle, Left VF vs Right Handle, Left VF	*t*(94) = .495, *p* = .622, *d* = .052
Right Handle Left VF vs Right Handle Right VF	*t*(94) = .999, *p* = .320, *d* = .103

VF, visual field

Next, we determined whether handedness, advertisement type, or handle orientation influenced how participants examined the handle of the product on the advertisement using a 2 x 2 x 2 mixed model ANOVA. There were main effects of handle orientation, *F*(1,91) = 39.948, *p* < .001, $\eta_{p}^{2}$ = .305, and advertisement type, *F*(1,91) = 35.381, *p* < .001, $\eta_{p}^{2}$ = .280. In keeping with our suggestion in relation to the text on the advertisements, participants fixated upon the object’s handle earlier when the advertisements did not contain text (*M* = 3.64 s, *SD* = 1.48) compared to when they did (*M* = 5.55 s, *SD* = 1.74). Interestingly, the 3 way interaction was significant, *F*(1,93) = 4.004, *p* = .048, $\eta_{p}^{2}$ = .042.

In order to understand the 3-way interaction, we split the data by advertisement type and conducted two additional 2 x 2 ANOVAs. For advertisements that contained text, we observed a main effect of handle orientation, *F*(1,45) = 51.759, *p* < .001, $\eta_{p}^{2}$ = .535. In contrast to the visual field data, participants fixated upon the right handle (*M* = 4.24 s, *SD* = 1.36) earlier than the left handle (*M* = 6.87 s, *SD* = 2.11). The main effect of handedness, *F*(1,45) = .029, *p* = .867, $\eta_{p}^{2}$ = .001, and the interaction, *F*(1,45) = .831, *p* = .367, $\eta_{p}^{2}$ = .018, were both non-significant. For advertisements where the text was removed, both main effects were non-significant: handle orientation, *F*(1,46) = .123, *p* = .728, $\eta_{p}^{2}$ = .003; handedness, *F*(1,46) = .648, *p* = .425, $\eta_{p}^{2}$ = .014. Importantly, the interaction was significant, *F*(1,46) = 5.302, *p* = .026, $\eta_{p}^{2}$ = .103, indicating that handedness influenced the way in which participants initially fixated upon the object handle. Although the pattern of the data is consistent with participants initially fixating upon the handle that was congruent with their own handedness, both contrasts were non-significant (*p*’s > .082).

#### Total fixation duration

As above, none of the effects in relation to advertisement type were significant, and we removed this factor to increase our power. The main effect of visual field was not significant, *F*(1,93) = .390, *p* = .534, $\eta_{p}^{2}$ = .004; however, handle orientation interacted with visual field, *F*(1,93) = 6.256, *p* = .014, $\eta_{p}^{2}$ = .063. Remaining effects were non-significant (Table 2), indicating handedness did not influence total fixation duration. The interaction of visual field and handle orientation showed that total fixation duration was longer in the left visual field when the handle was located on the left as compared to when it was located on the right, *t*(94) = 3.166, *p* = .002, *d* = .325. Remaining comparisons were non-significant (Table 2).

**Table 2:** Statistics for total fixation duration.

Effect	Statistic
Handle Orientation	*F*(1,93) = 3.463, *p* = .066, $\eta_{p}^{2}$ = .036
Handedness	*F*(1,93) = .123, *p* = .726, $\eta_{p}^{2}$ = .001
Handle Orientation*Handedness	*F*(1,93) = .048, *p* = .827, $\eta_{p}^{2}$ = .001
VF*Handedness	*F*(1,93) = .052, *p* = .820, $\eta_{p}^{2}$ = .001
Handle*VF*Handedness	*F*(1,93) = 1.163, *p* = .284, $\eta_{p}^{2}$ = .012
Left Handle, Left VF vs Left Handle, Right VF	*t*(94) = 1.457, *p* = .148, *d* = .150
Right Handle, Left VF vs Right Handle, Right VF	*t*(94) = .455, *p* = .650, *d* = .047
Left Handle, Right VF vs Right Handle, Right VF	*t*(94) = 1.231, *p* = .221, *d* = .126

VF, visual field

We then conducted an analysis to determine whether handedness, handle orientation, and advertisement type influenced total fixation duration on the product handle. The main effects of handle orientation: *F*(1,91) = .548, *p* = .461, $\eta_{p}^{2}$ = .006, and handedness: *F*(1,91) = .905, *p* = .344, $\eta_{p}^{2}$ = .010, were non-significant. All interactions were also non-significant: handle orientation x advertisement type, *F*(1,91) = .774, *p* = .381, $\eta_{p}^{2}$ = .008; handle orientation x handedness, *F*(1,91) = .190, *p* = .664, $\eta_{p}^{2}$ = .002; handedness x advertisement type, *F*(1,91) = 2.280, *p* = .135, $\eta_{p}^{2}$ = .024; handle orientation x handedness x advertisement type, *F*(1,91) = .164, *p* = .686, $\eta_{p}^{2}$ = .002. Although eye movements toward the object handle did not differ based on handedness or handle orientation, the main effect of advertisement type was highly significant, *F*(1,91) = 57.001, *p* < .001, $\eta_{p}^{2}$ = .385. Total fixation duration upon the product handle, across all stimuli, was significantly longer when the advertisements did not contain text (*M* = 16.69 s, *SD* = 6.07), as compared to when they did (*M* = 8.95 s, *SD* = 3.67).

#### Fixations before

The main effects of handle orientation, *F*(1,91) = 5.082, *p* = .027, $\eta_{p}^{2}$ = .053, visual field, *F*(1,91) = 16.449, *p* < .001, $\eta_{p}^{2}$ = .153, and advertisement type, *F*(1,91) = 5.274, *p* = .024, $\eta_{p}^{2}$ = .055, were all significant. In keeping with the total fixation duration data, participants performed, on average, 8.63 (*SD* = 2.45) fixations prior to examining the handle when the advertisements did not contain any text. In contrast, an average of 18.89 (*SD* = 4.47) fixations were made before fixating upon the handle when the advertisements did contain text. Importantly, the interaction of handle orientation and visual field was also significant, *F*(1,91) = 31.402, *p* < .001, $\eta_{p}^{2}$ = .257. Remaining effects were non-significant (Table 3), indicating that once again handedness did not influence eye movements.

**Table 3:** Statistics for fixations before.

Effect	Statistic
Handedness	*F*(1,91) = 3.674, *p* = .058, $\eta_{p}^{2}$ = .039
Handle Orientation*Handedness	*F*(1,91) = .177, *p* = .675, $\eta_{p}^{2}$ = .002
Handle Orientation*Ad Type	*F*(1,91) = .214, *p* = .645, $\eta_{p}^{2}$ = .002
Handle Orientation*Handedness*Ad Type	*F*(1,91) = .045, *p* = .832, $\eta_{p}^{2}$ < .001
VF*Handedness	*F*(1,91) = .097, *p* = .756, $\eta_{p}^{2}$ = .001
VF*Ad Type	*F*(1,91) = 2.986, *p* = .087, $\eta_{p}^{2}$ = .032
VF*Handedness*Ad Type	*F*(1,91) = .084, *p* = .772, $\eta_{p}^{2}$ = .001
Handle Orientation*VF*Ad Type	*F*(1,91) = 1.979, *p* = .163, $\eta_{p}^{2}$ = .021
Handle Orientation*VF*Handedness	*F*(1,91) = .591, *p* = .444, $\eta_{p}^{2}$ = .006
Handle Orientation*VF*Handedness*Ad Type	*F*(1,91) = .046, *p* = .831, $\eta_{p}^{2}$ = .001

VF, visual field

Paired-samples *t*-tests showed that participants changed their fixation pattern based on handle orientation. When the object handle was located on the left side, participants performed an average of 4.22 fixations in the left visual field (*SD* = 1.62), compared to 6.03 fixations in the right visual field (*SD* = 2.05) prior to examining the object handle, *t*(94) = 6.455 *p* < .001, *d* = .665. Similarly, participants performed an average of 4.95 fixations in the right visual field when the handle was on the right side (*SD* = 1.87) compared to 6.03 fixations in the right visual field when the handle was on the left side (*SD* = 2.05) prior to examining the object handle, *t*(94) = 6.373, *p* < .001, *d* = .655.

## Discussion

Investigation into how affordances influence preferences ratings and visual scan patterns of advertisements has been limited. Furthermore, although prior research [11, 12] has examined how object affordances influence preference ratings and purchase intention, these studies have focussed predominantly on right-handers. We built upon this prior work by actively recruiting left-handers, including a large number of advertising stimuli, and also assessing perceived value using a novel rating scale. To determine whether preferences could be explained by differences in the spread of attention across the visual field and object affordances, we included three dependent eye tracking variables as an objective measure of spatial attention.

Time to first fixation data revealed that, overall, fixations within the left visual field occurred earlier. More interestingly, our data indicated that the location of the object handle influenced time to first fixation. Namely, participants fixated the left visual field sooner when the object’s handle was on the left side, whereas the right visual field was fixated earlier when the handle was oriented toward the right. These data demonstrate that the orientation of the object handle influenced the way in which participants initially fixated upon the images. Importantly, we must note that time to first fixation was not influenced by handedness; all participants fixated upon the side of the advertisement with the handle. As such, there appears to be a difference in the ability of the object handle to attract and capture attention.

We also examined how many fixations participants made prior to examining the handle (i.e., fixations before). Further illustrating that the location of the object handle influenced the distribution of attention, participants made fewer fixations within the left visual field prior to examining the handle, when the object handle was located on that side. Similarly, participants made fewer fixations within the right visual field prior to examining the handle, when the handle was on the right as compared to the left. These findings provide further evidence of the attention-grabbing properties of the object handle.

Our final measure of objective attention showed that total fixation duration within the left visual field was modulated by the location of the object handle. Namely, participants spent more time examining the left visual field when the handle was on the left, compared to when the handle was on the right. This finding illustrates that while object handles attracted attention by influencing time to first fixation when the handle was on the left, they also influenced attention retention and led participants to fixate the left side longer. As this finding was not influenced by handedness, the positioning of the handle on the left side could have increased the retention of attention across all participants, because viewing objects with left side affordances is simply more novel.

Whether participants were aware or not, eye movement patterns show some level of behavioural modification in keeping with the affordance of the object–participants fixated the side with the handle more quickly, and for a longer period of time, regardless of their own handedness. Potentially, this behavioural change reflects an automatic process that initiates preparation for an interaction with an object. As can be seen in the heat maps in Fig 1, participant attention was drawn towards the action-performing or functional area of the object as opposed to the handle [17]. Prior research has shown that individuals tend to focus their fixations between the graspable area and the object’s centre of mass [37]. However, precise fixation locations appear to be dependent upon the task participants are asked to perform. For example, Belardinelli, Herbort, and Butz [38] found that fixations were focused on the object handle when participants were asked to simulate lifting the object, and were focused on the centre of mass when participants were asked to classify the object (see Papies, Best, Gelibter, and Barsalou [39], who highlight the importance of simulating interaction). Future research should make use of eye tracking while participants are asked to imagine interacting with the object to determine whether fixations upon the handle in advertisements are more numerous in this instance. It could also be the case that handedness influences eye movements only when participants are imagining object interaction (see [27]).

We also manipulated whether or not participants viewed advertisements with text in our laboratory sample. Indeed, prior research suggests that while pictorial elements attract the most attention, attention is also necessarily devoted to the text on advertisements [40]. We included text on the advertisements to increase their ecological validity, but the text could have actually distracted participants and led them to spend less time examining the objects themselves. As one might expect, participants fixated handles earlier and spent more time examining the handles of the objects when no text was present on the advertisement. Furthermore, participants made significantly more fixations upon the advertisements prior to examining the handle when the advertisement contained text than when it did not. As it was no longer necessary to disengage and transfer attention from the advertised product to the text [40], participants were able to examine the products themselves more thoroughly, including the object handle (i.e., our area of interest).

Intriguingly, we also found that participants indicated they were more likely to purchase the products that were advertised without text, than those that appeared alongside text. Prior research has shown that texts that engage narrative processing [41] and allow for multiple interpretations [42] are preferred and are inherently pleasurable to readers. As such, it is possible that participants did not find the text on our advertisements to be sufficiently interesting or pleasurable, and therefore the advertisements with text were less effective in eliciting a positive future purchase intention. It is also possible that the absence of text encouraged participants to examine the advertised objects more thoroughly and therefore they felt more inclined to purchase the items. We must acknowledge that only a subset of our participants viewed the advertisements without text and all of these participants performed the experiment in the laboratory, because we believed the text might impact upon eye movement behaviour. Given our findings suggest that ratings data might also be influenced by the presence of text, this factor should be explored in further detail in the future. Further research, which examines why participants prefer advertisements without text is required to test these propositions.

Although we observed higher ratings for aesthetic appraisal when advertisements depicted left handled objects, this finding was driven by our left-handed participants. Left-handers showed a significant preference for objects that afforded the left hand, whereas, right-handers gave similar ratings for advertisements of left and right handled objects. Data for purchase intention mirrored our findings for aesthetic appraisals as left-handers also indicated they were more likely to purchase products that afforded the left hand compared to identical products oriented rightward. Although right-handers showed a slightly greater intent to purchase products with a rightward affordance, this difference was not significant. Finally, left-handers attributed products with a left-facing handle a higher monetary value than identical products with a right-facing handle. Once again, right-handed individuals attributed a similar perceived value to both advertisements.

It is interesting that left-handers showed these unique preferences compared to right-handers, even though they did not fixate left handled objects earlier or more frequently than objects oriented rightward. Indeed, eye tracking data failed to show any influence of handedness on objective attention in relation to either attention-grabbing and attention retention. It is possible that participants were aware of the orientation of the handle and that they noticed that the location of the handle varied across the advertisements. Furthermore, given our participants were given an unlimited amount of time to provide their ratings, preference decisions likely reflect seemingly conscious decisions. Indeed, it is possible that participants used handle orientation to inform their preference ratings. As we did not query participants about the reasoning behind their preferences, we cannot be sure about this suggestion. Furthermore, findings from implicit social cognition [43, 44] argue that this line of inquiry would not necessarily provide us with accurate information regarding preference decisions. Instead, we suggest that left-handers’ preferences for left affordances could be implicit or subconscious.

Implicit social cognition [43] provides a framework for understanding the cognitive processes that underlie our attitudes and judgment without our conscious awareness. Only a small amount of information reaches our consciousness at any given moment [44]; although some of this information might be relevant to our current thoughts or behaviours, our ability to identify causality between our thoughts and our subsequent judgments is far from perfect [45–49]. Indeed, as stated by Nosek et al. [44], reported causes of behaviour do not necessarily have any relationship with the true reasons for behaviour [47]. Given the inaccuracy of self-report and research showing that people often report incorrect reasons for their judgments and behaviours [45–49], we are suggesting that the reported preferences for left affordances by left-handers are likely the result of information that is not subject to conscious awareness.

Our findings in relation to right-handed individuals are inconsistent with prior research, wherein right-handers preferred objects that were oriented toward their dominant hand [11–13, 50]. However, more recent research has failed to replicate these findings in relation to advertising and purchase intention. For example, Pecher and van Dantzig [27] found, across eight experiments, that handedness and product orientation did not interact to influence intention to purchase. As noted by Pecher and van Dantzig [27], the majority of this prior research did not make use of advertising stimuli [13, 50], or used a very limited number of stimuli [12]. Our findings in relation to right-handers are consistent with Pecher and van Dantzig [27], support the position that right-handers tend to pay less attention to object orientation [11], and suggest that right-handed people are indeed less aware of the impact that object affordances play in our everyday lives.

Interestingly, we did observe a strong preference amongst left-handers to find advertisements of objects with their handle on the left side more aesthetically pleasing. It appears that the relationship between handedness and object preferences [11, 12] extends to aesthetics, purchase intention and perceived value ratings of advertisements amongst left-handers only. Our findings here stand in contrast to Pecher and van Dantzig [27], who observed one significant effect, which was a right handle preference amongst left-handers. Living in a right-handed world has likely decreased the need for right-handers to notice the location of an object’s handle, whereas left-handers are more acutely aware of object orientation and affordances as they have been forced to adapt to right-handed preferences. As such, left-handed people might place more value on product orientation when making purchase decisions. An observational study, which examines the frequency with which objects are presented with their handles on the left or the right side in supermarkets and shops could be an interesting avenue for future research as it would shed light on this suggestion.

We varied the location of the label anchors for our rating scales as prior research has shown that participants respond more positively when positive labels are located on the left side on both Likert-type and visual analogue scales [30, 51]. Given we included this manipulation to control for any possible effects, we did not make any hypotheses about this factor and therefore did not include it in our primary analysis. Interestingly, our exploratory analysis suggests that this factor did not influence our results, which suggests that although the location of the label anchors can influence participant responses, this influence does not occur on every occasion. It remains possible that handedness and location of label anchor interact to influence rating data, which could potentially be investigated in the future.

Given our findings are not consistent with either Elder and Krishna [12] or Pecher and van Dantzig [27], it is important to consider why all three sets of findings differ. One possible explanation relates to the ratings scale. We used a visual analogue scale, which allowed participants to respond along a continuous scale, rather than using a Likert-type scale [12, 27]. It is possible that the use of a restrictive categorial scale did not allow participants to indicate slight preference differences, but instead encouraged them to choose the similar categories across trials, particularly if they did not have any strong preferences [52]. Visual analogue scales show high reproducibility and, importantly, sensitivity, which makes them an excellent option for preference judgments [53]. Furthermore, it is possible that our right-handed participants did not show the same preferences as those reported by Elder and Krishna [12], because our participants completed 20 trials, whereas theirs completed only 2 trials (one for each handle orientation).

Given the methodological differences between our study, Pecher and van Dantzig [27], and Elder and Krishna [12], all conclusions to date must be interpreted with caution. We believe these effects are interesting, but also acknowledge that our data invite the opportunity for future research in relation to how handedness and handle orientation interaction to influence purchase decisions. Indeed, a collaborative laboratory-based study, which provides the opportunity to recruit a large number of left-handed participants in person would be a good way to shed further light on the current data.

Another important consideration relates to our data collection methods. Given it is more difficult to recruit left-handed participants in the lab, particularly at small universities, we collected additional ratings data online, where we were able to access a larger sample of left-handed participants. We acknowledge that this recruitment tactic resulted in a combined sample of both laboratory and online participants. However, this approach was necessary to obtain a sufficiently large sample, in line with our a priori power calculation. Furthermore, we do not have any reason to believe that the ratings data we collected online would differ in a systematic way from the data we collected in the lab. Future research should replicate the design we have used here with a larger laboratory sample to confirm our findings.

Intriguingly, our findings also suggest that left-handers are prepared to pay more money for products that afford the left hand and are therefore easier to interact with. Left-handers might genuinely have more experience in purchasing products that are specifically designed for left hand use, and could have previously paid a premium price for an item that was specifically designed for left hand use. However, it could also be the case that these individuals are not consciously aware that handle orientation influences future purchase intention and perceived value when viewing advertising materials. As such, it would be interesting to query left-handers further in future research, to determine which factors they considering in making their ratings.

Other research into handle alignment effects comprise stimulus-response compatibility paradigms in which a lateralised, speeded response is made to object stimuli [54]. Some accounts of these stimulus-response compatibility effects attribute them to a simple location coding mechanism, rather than affordance-related motor activation (see Proctor and Miles [54] for a discussion). Location coding explanations argue that an object’s handle is a salient, asymmetrical feature, which can produce a speed/accuracy advantage for a spatially compatible response, as in a classic Simon effect [55]. However, this alternative explanation for alignment effects is not relevant to the current study, which examined preference judgements rather than speeded, lateralised responses or reactions to objects. Furthermore, given that we counterbalanced locations of the anchor labels on our ratings scales, any location coding effects were eliminated.

Previous research points toward the automatic activation of affordances when viewing images of graspable objects [12] (see Grèzes and Decety [56] for a review of relevant neuroimaging research). Arguably, right-handers might have stronger automatic activations for their dominant hand as a result of living in a right-hand world, where grasping affordances suit them better and left-handers are forced to be more flexible. In support of this suggestion, our correlational data show that preferences are positively correlated with strength of handedness, such that more strongly left-handed participants showed an increased preference for advertisements of left-handled objects.

The premise underlying this suggestion is that congruency between grasping affordance and dominant hand facilitates interaction and creates a preference over time for the observer. In turn, the fluency of these interactions is likely more pronounced amongst left-handers, because of their novelty. By contrast, right-handers are likely to have a higher number of easy and fluent interactions with right-handed objects. The mechanism proposed here differs from affordance accounts of stimulus-response compatibility effects described above, which require a stronger activation of affordance-related motor programs when making a speeded response to some other feature of the viewed object [57–59]. Instead, we suggest that the underlying affordance activation facilitates interactions with objects with congruent handles in the real world, which leads to the preferences we have reported here.

As a consequence of very real danger when using certain right-handed products (e.g., scissors, circular saws), it is possible that left-handers genuinely prefer the sight of a product that is easier for them to interact with. Importantly, our data suggest that these preferences extend to everyday products that are not genuinely handed (i.e., iron, kettle, saucepan). In contrast, right-handers likely take their own handedness for granted, and therefore fail to devote as much attention to object affordance. Consistent with our expectation, the novel “perceived monetary value” scale revealed that left-handers gave left-handed products a higher perceived value. Potentially, left-handers indicated these products were worth more money because they have direct experience in purchasing these products, which indeed are more expensive than the equivalent right-handed ones. It could also be the case that the ease with which left-handers can use leftward oriented products is appealing and therefore they perceive these products to be worth the extra money to them personally. Further, our failure to observe any handedness-based differences in scan patterns, means that participant preferences were altered despite the fact that participants had gathered information about the products in the same fashion. Importantly, our findings have important consequences for the marketing of left-handed products, as left-handers are aware (either consciously or subconsciously) of object affordances and significantly prefer products that more easily afford action.

## Supporting information

S1 Text Exploratory analysis of location of label anchors based on the orientation of the handle for each rating scale. (DOCX)
