# Supplementary material for: Left-handers know what’s left is right: Handedness and object affordance
Source: PLoS One. 2019 Jul 24;14(7):e0218988. doi: 10.1371/journal.pone.0218988 (PMC6655602; doi:10.1371/journal.pone.0218988)
Supplement: S1 Text — (DOCX) [file pone.0218988.s001.docx]

Supplementary Material

Nicole A. Thomas, Rebekah Manning, Elizabeth J. Saccone

**Exploratory analysis**

We conducted 2 (handle orientation: left, right) x 2 (location of label anchor: left, right) mixed ANOVAs for each rating scale: aesthetic appraisal, purchase intention, and perceived value to explore whether the location of the label anchors influenced participant ratings. We did not include handedness in this analysis as we controlled the location of the label anchors such that half of all participants completed each version of the task; however, we did not account for participant handedness as we did not plan to analyse this factor.

**Aesthetic appraisal**

The main effect of location of label anchor, *F*(1,265) = 1.541, *p* = .216, $\eta_{p}^{2}$ = .006, was non-significant, indicating that ratings for aesthetic appraisal did not differ based location of label anchor. Furthermore, the interaction between handle orientation and location of label anchor was non-significant, *F*(1,265) = 1.428, *p* = .233, $\eta_{p}^{2}$ = .005. The main effect of orientation was significant, *F*(1,265) = 12.909, *p* < .001, $\eta_{p}^{2}$ = .046; advertisements wherein products had a leftward oriented handle were rated as more aesthetically pleasing than those with a rightward handle.

**Purchase intention**

The main effect of location of label anchor, *F*(1,265) = .356, *p* = .551, $\eta_{p}^{2}$ = .001, was non-significant, indicating that purchase intention did not differ based location of label anchor. Furthermore, the interaction between handle orientation and location of label anchor was non-significant, *F*(1,265) = .786, *p* = .376, $\eta_{p}^{2}$ = .003. The main effect of orientation was significant, *F*(1,265) = 9.638, *p* = .002, $\eta_{p}^{2}$ = .035; participants indicated they were more likely to purchase products with a left handle orientation than products with a right handle orientation in the advertisement.

**Perceived monetary value**

The main effect of location of label anchor, *F*(1,265) =.051, *p* = .822, $\eta_{p}^{2}$ < .001, was non-significant, indicating that perceived monetary value did not differ based location of label anchor. Furthermore, the interaction between handle orientation and location of label anchor was non-significant, *F*(1,265) = 1.241, *p* = .266, $\eta_{p}^{2}$ = .005. The main effect of orientation was significant, *F*(1,265) = 4.658, *p* = .032, $\eta_{p}^{2}$ = .017; participants indicated that products with a leftward oriented handle were more valuable than those with a rightward handle.
